# Parrotfish Size: A Simple yet Useful Alternative Indicator of Fishing Effects on Caribbean Reefs?

**DOI:** 10.1371/journal.pone.0086291

**Published:** 2014-01-20

**Authors:** Henri Vallès, Hazel A. Oxenford

**Affiliations:** 1 Department of Biological and Chemical Sciences, The University of the West Indies, Cave Hill Campus, Cave Hill, Barbados; 2 Centre for Resource Management and Environmental Studies (CERMES), The University of the West Indies, Cave Hill Campus, Cave Hill, Barbados; James Cook University, Australia

## Abstract

There is great need to identify simple yet reliable indicators of fishing effects within the multi-species, multi-gear, data-poor fisheries of the Caribbean. Here, we investigate links between fishing pressure and three simple fish metrics, i.e. average fish weight (an estimate of average individual fish size), fish density and fish biomass, derived from (1) the parrotfish family, a ubiquitous herbivore family across the Caribbean, and (2) three fish groups of “commercial” carnivores including snappers and groupers, which are widely-used as indicators of fishing effects. We hypothesize that, because most Caribbean reefs are being heavily fished, fish metrics derived from the less vulnerable parrotfish group would exhibit stronger relationships with fishing pressure on today’s Caribbean reefs than those derived from the highly vulnerable commercial fish groups. We used data from 348 Atlantic and Gulf Rapid Reef Assessment (AGRRA) reef-surveys across the Caribbean to assess relationships between two independent indices of fishing pressure (one derived from human population density data, the other from open to fishing versus protected status) and the three fish metrics derived from the four aforementioned fish groups. We found that, although two fish metrics, average parrotfish weight and combined biomass of selected commercial species, were consistently negatively linked to the indices of fishing pressure across the Caribbean, the parrotfish metric consistently outranked the latter in the strength of the relationship, thus supporting our hypothesis. Overall, our study highlights that (assemblage-level) average parrotfish size might be a useful alternative indicator of fishing effects over the typical conditions of most Caribbean shallow reefs: moderate-to-heavy levels of fishing and low abundance of highly valued commercial species.

## Introduction

The integrity of coral reefs across the Caribbean region is under great threat. Live coral cover has declined considerably across the region since the late 1970s [1], [2] and so has the production of the calcium carbonate reef framework [3] that is host to a great diversity of reef organisms. Concomitant with these changes, there have been region-wide decreases in structural complexity of coral reefs [4] and increases in macro-algae [2], [5]. This trend in habitat degradation has likely contributed to region-wide decreases in the abundance of the reef fish populations [6], already depressed from historic and current high levels of fishing pressure [7]–[9]. All this raises serious concerns about the future of Caribbean coral reefs and, consequently, about the capacity of small island developing states of the Caribbean to adapt to the effects of climate change [7], [8], [10], given their current heavy reliance on the ecosystem services provided by reefs. For example, it is estimated that fishing directly employs more than 120,000 fishers in the Caribbean [11], with coral reef fisheries specifically yielding net annual economic benefits of nearly $ US 400 million [7], and that coral-reef related tourism in the region generates annual net benefits of about $ US 2.7 billion [7].

On-going coral reef degradation is being caused by interacting stressors operating at both broad (ocean warming and acidification; sea level rise) and local (overfishing; sedimentation; nutrient run off; species invasion; storm damage; disease; algal blooms) scales [12]. In the short term, little can be done to reduce the direct effect of global stressors on coral reefs. Thus, considerable emphasis has been placed on outlining management measures seeking to mitigate the effect of human-induced local stressors, with the hope of increasing the resilience of coral reefs to global ones [12], [13]. Although some of the debate has highlighted discrepancies between what are considered to be the main local factors driving coral decline [2], [14]–[16], it is widely recognized that overfishing will hamper the recovery of coral reefs because it reduces the capacity of fish herbivores to effectively graze the algae that compete with corals for space [17]–[19]. Thus, management measures aimed at reducing fishing pressure on coral reefs are an obligate component of a larger strategy to reverse the current trend in reef degradation [2], [12], [13], [20].

A recent study indicates that almost 70% of coral reefs across the Caribbean region are under great threat of overfishing [7], highlighting the urgent need for more effective fisheries management. A key component of successful fisheries management is the development of reliable indicators of fishing effects [21], [22]. Considerable work has focused recently on developing indicators that capture key attributes of the structure and function of entire fished communities in the context of ecosystem-based fisheries management (e.g. [23]–[27]). Fish metrics aggregating attributes of multiple co-existing species will tend to be more robust than population-level metrics to natural variability in species-specific factors that are not associated with fishing [23] as well as sampling methodology [28]. This approach seems particularly well suited to the multi-species and multi-gear small-scale fisheries that prevail in the shallow coral reefs of the Caribbean [29]. In theory, the effectiveness of such an indicator will depend largely on its ability to separate fishing effects from those of natural variation over the spatial and temporal scales that are relevant to managers [21]. In practice, it is increasingly recognized that successful fisheries management will hinge upon the development of indicators that are simple enough to be effectively interpreted and communicated across the different stakeholder groups involved in decision-making (managers, policy makers, fishers, general public) [26], [27], [30]. Further, such indicators should be relatively easy to measure, given the limited resources available to reef managers in the Caribbean [31]. Overall, this means an unavoidable trade-off among the different valued properties of any indicator [22].

Fish metrics describing aggregate attributes (e.g. average individual size, total biomass, total numerical abundance) of groups of commercially valued carnivore species such as snappers and groupers are widely-used as simple and intuitive indicators of fishing effects in coral reefs (e.g [32]). This is because the life history characteristics of these commercial species, i.e. large body size, slow growth and late maturity, make them highly vulnerable to the effects of fishing [33], [34]. There is considerable empirical evidence showing that fish metrics based on these commercial fish groups predictably decrease with increasing fishing pressure at both local (e.g. [35], [36]) and broad spatial scales (e.g. [37]). Inherently, these metrics also provide information about the integrity of the trophic structure of the reef fish community, which can be compared with potential baselines (e.g. [38], [39]).

Paradoxically, the high vulnerability of the commercially-valued carnivore fish species that make up these fish metrics might limit the usefulness of the latter as indicators of fishing effects over the conditions that currently characterize most Caribbean reefs: pervasive high levels of fishing pressure [7]. This is so because these highly valued commercial fishes are likely to become relatively rare under moderate to high levels of fishing pressure and take a long time to recover after cessation of fishing (e.g. [36]). This implies that our ability to distinguish between moderately and heavily fished reefs using metrics based on these commercial fish groups is likely to be compromised, especially with limited monitoring capacity, as is often the case in the Caribbean [31]. By extension, the impact of management measures aimed at reducing fishing effects in heavily fished reefs might be more difficult to detect when using metrics based on commercial fish groups. Under such conditions, metrics derived from fish groups comprising less vulnerable fished species (i.e. those with smaller body size, faster growth and earlier maturity) might be more reliable indicators of fishing effects because they are expected, *ceteris paribus*, to have a less drastic response to fishing and therefore to provide the minimum critical biomass upon which the effects of fishing can be measured with higher precision.

One fish group with considerable potential as an alternative indicator of fishing effects in the shallow reefs of the Caribbean is the parrotfish family (Scaridae, Suborder: Labroidei [40]). With 16 species in the Caribbean, this family of herbivores is dominated by species of the *Sparisoma* and *Scarus* genera (14 species), most of which are found throughout the region [41]. Parrotfishes are fished in many locations of the Caribbean [42]–[44] and in some Caribbean fisheries they dominate the catch [45], [46]. The latter is not surprising given that the total biomass of most of the major fish families vulnerable to fishing is currently dominated by parrotfish biomass in most shallow locations throughout the Caribbean (Table 1). In most such locations, parrotfish biomass exceeds the combined biomass of snapper and grouper families as well as the combined biomass of selected highly valued commercial species that are typically used as indicators of fishing effects (Table 1). Considering the evidence that carnivore biomass should be the most important component of fish biomass in relatively undisturbed sites [38], [39], the latter supports the idea of pervasive effects of intense fishing throughout the Caribbean region leading to fishing down the food-web [47]. It also suggests that parrotfishes, as a family, are more resilient to the effects of intensive fishing and might therefore consistently provide the critical minimum biomass upon which variability in fishing effects can be measured with high precision across the region.

**Table 1:** Percentage of Atlantic and Gulf Rapid Reef Assessment (AGRRA) fish surveys in 17 state/territories (as referred to by AGRRA) carried out in different coral reef habitats between 1997 and 2004 where parrotfishes were the dominant family in terms of biomass and where parrotfish biomass exceeded that of highly valued commercial fish groups.

State/Territory	Fish Surveys (n = 641)	PAR is dominant family	PAR>SNP+GRP	PAR>COM
Bahamas	41	63.4	82.9	75.6
Belize	46	84.8	82.6	69.6
Cayman Islands	40	80.0	87.5	77.5
Costa Rica	3	33.3	100.0	66.7
Cuba	162	45.1	67.3	54.9
Dominican Republic	33	42.4	90.9	72.7
Jamaica	60	81.7	100.0	95.0
Mexico	34	38.2	64.7	47.1
Netherland Antilles	28	60.7	100.0	92.9
Nicaragua	14	35.7	35.7	42.9
Panama	43	86.0	95.3	88.4
Puerto Rico	17	82.4	100.0	88.2
St Vincent	5	100.0	100.0	80.0
Turks and Caicos	27	77.8	74.1	51.9
USA (Florida)	53	39.6	67.9	50.9
Venezuela	13	84.6	76.9	84.6
Virgin Islands	22	72.7	81.8	50.0
	Median	72.7	82.9	72.7

Parrotfish biomass (PAR), snapper biomass (SNP) and grouper biomass (GRP), and combined biomass of a selection of species (COM) considered by AGRRA to be “Commercially significant”, including snappers, groupers, grunts, triggerfishes and large labrids.

Thus, metrics derived from parrotfish assemblages potentially constitute a viable alternative or complement to metrics derived from commercial fish groups as indicators of fishing effects in the Caribbean. Unlike the surgeonfishes, the other major family of herbivores vulnerable to fishing, Caribbean parrotfishes exhibit considerable species diversity and comprise species that differ considerably in life history traits and body length (i.e. up to one order of magnitude) [41], [48]. This implies that different parrotfish species will differ in their vulnerability to the size-dependent effects of fishing and that such effects might be detectable using different assemblage-level metrics (e.g. [25]). In the Indo-Pacific, there is considerable consistent evidence of the effects of fishing on parrotfishes at both the population and assemblage level; such effects include reductions in the abundance, biomass and/or average size of the larger parrotfish species as well as shifts in the size-structure of entire parrotfish assemblages through the decline of large individuals [49]–[51]. In line with this evidence, Clua and Legendre [52] formerly highlighted the potential of parrotfishes as a family to reveal gradients of fishing pressure in the South Pacific through a combination of both a reduction in fish size of individual species and shifts in species dominance driven by size-dependent vulnerability to fishing. There is evidence that parrotfishes might also help reveal gradients of fishing pressure in the Caribbean. In particular, Hawkins and Roberts [53] showed a negative relationship between average parrotfish size and fishing pressure for several parrotfish species and also showed size-dependent changes in the abundance of Caribbean parrotfish species that were likely driven by fishing. However, despite their apparent potential, we are not aware of any study specifically focusing on the usefulness of metrics derived from parrotfishes as indicators of fishing effects. Importantly, parrotfishes play key roles on the reef as algal grazers [13], [54] and bio-eroders [55]. This implies that metrics derived from parrotfishes not only have the potential to provide valuable information about variability in fishing effects, they will also inform about the state of this key functional group, thus possibly more effectively linking fishing effects to the grazing and bio-erosion functions that are critical to reef health (e.g. [49]).

In this study we specifically evaluate the potential of different metrics derived from parrotfish assemblages to inform about variability in fishing effects in the Caribbean region. We deliberately focus on three simple, assemblage-level, aggregate fish metrics, i.e. average fish weight (an estimate of average individual fish size), fish biomass and fish density, because these metrics are intuitive and likely to be effectively communicated and interpreted within and among different stakeholder groups [26]. As part of this assessment, we compare the performance of the parrotfish metrics with the same metrics derived from fish groups of co-occurring, highly valued, commercial carnivore species, including snappers and groupers, which we here consider as our baseline indicators We hypothesize that because Caribbean reefs are typically moderately to heavily exploited, parrotfish metrics will be more effective at capturing variability in fishing effects and will therefore exhibit stronger relationships with fishing pressure than metrics derived from highly valued commercial fish groups, within and across locations throughout the Caribbean region.

## Methods

### Data-Sets

We carry out this metric evaluation and comparison by examining the strength of the relationships between fish metrics obtained from reef fish community surveys across the Caribbean region and two independent indices of fishing pressure; an approach consistent with that typically used to evaluate fish community indicators for fisheries management [56], [57]. Indeed, for a fish metric to be considered as a potential indicator of fishing effects, it must exhibit sensitivity to variability in fishing pressure [22], [56], [57]. By using two independent indices of fishing pressure, we thus evaluate the robustness of this sensitivity. To shed some light on the factors driving variability in the most promising parrotfish metrics identified, we further investigated associations between one of the indices of fishing pressure and variability in both the average fish weight and relative fish density of the individual parrotfish species making up the parrotfish assemblages.

#### Fish metrics

To obtain data on reef fish communities across the Caribbean region, we used a large fisheries-independent data-set readily available to the general public – the Atlantic and Gulf Rapid Reef Assessment (AGRRA) data-set (http://www.agrra.org/), which is based on a standardized sampling protocol and includes fish surveys covering most of the region. Briefly, the fish sampling protocol consists in divers swimming along a number of haphazardly laid 2×30 m belt transects while identifying individual fishes and allocating them into one of 6 length intervals, with all fish recorded generally being >5 cm in total length. The number of transects varies across locations and in a few locations transects are 50 m long.

The AGRRA data include (species and family level) fish density and biomass estimates for the most important reef fish groups vulnerable to fishing, including parrotfishes, snappers, groupers, grunts and large labrids among others. Fish biomass estimates are derived by adding individual fish weights found at a site. The latter are estimated using the body lengths of individual fish and the length-weight conversion W = aL^b^, where “W” is weight in grams, “L” is body length in cm (i.e. the mid-point of the length interval) and parameters “a” and “b” are constants available in the AGRRA data-set, which are based on values obtained from Fishbase (www.fishbase.org). We calculated the third metric, average fish weight, by simply dividing total fish biomass of a given fish group at a site by its corresponding number of fish counts.

**Figure 1:**
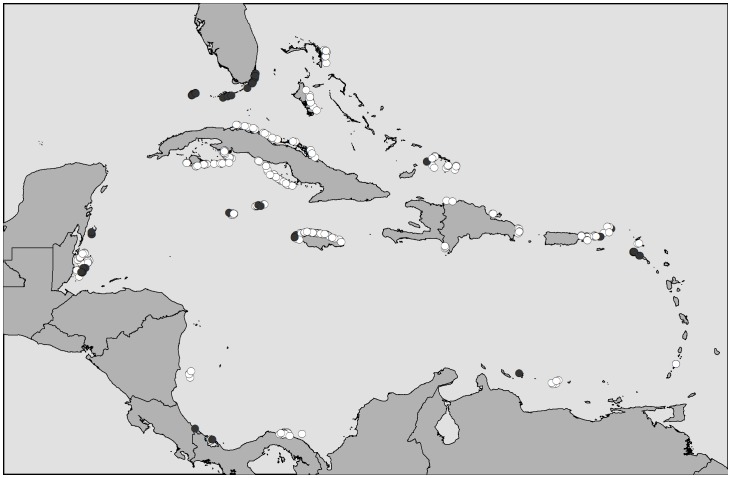
Location of 348 Atlantic Gulf Rapid Reef Assessment (AGRRA) surveys across the Caribbean region carried out between 1998 and 2004 and involving the use of ten 60 m^2^ transects. Red dots indicate sites that exhibit either effective full or partial protection against fishing. Green dots indicate sites that are either unprotected, or with ineffective protection, or of unknown protection effectiveness status. Fishing protection effectiveness categories are based on [7] and [60]. Table 2 provides additional information on the location of the sampling sites.

We selected a sub-set of the AGRRA data for our analyses. Because these data have been collected from different reef habitats (crest, patch reef, fore reef), we retained only those fish surveys carried out in the fore reef habitat (e.g. [15]) so as to minimize potentially confounding habitat-associated biases in our regional comparison. We subsequently retained data from those fish surveys in which exactly the same sampling effort had been deployed to characterize the fish communities, i.e. ten 2×30 m transects (600 m^2^ of reef area). This ensured that potential differences in precision associated with the fish metrics were not confounded by potential differences across locations in sampling effort, given that different locations would likely be subject to different levels of fishing pressure. We focused on those fish surveys carried out between 1998 and 2004, which resulted in a subset of 348 fish surveys that include locations across 17 states/territories (Figure 1; Table 2).

**Table 2:** Sampling batch ID, location, year code, average latitude and longitude (decimal degrees) coordinates and number (n) of Atlantic and Gulf Rapid Reef Assessment (AGRRA) surveys included in the analyses.

Batch ID	Location (year)	Latitude	Longitude	n
3	Bahamas (1998)	24.4078	−77.6589	13
6	Netherland Antilles (1999)	12.2183	−68.3528	1
9	Cayman (1999)	19.4808	−80.6992	17
10	St. Vincent (1999)	12.6331	−61.3496	3
13	Bahamas (1999)	26.4795	−76.9828	9
14	Turks and Caicos (1999)	21.5753	−71.7775	20
16	Belize (1999B)	17.2038	−87.5874	11
17	Venezuela (1999)	11.8175	−66.7459	5
18	Costa Rica (1999)	9.7500	−82.8058	1
19	Virgin Islands (1999)	18.3425	−64.7827	8
21	Netherland Antilles (1999)	17.5863	−63.1002	21
24	Cayman (2000)	19.7123	−79.8262	6
25	Virgin Islands (2000)	18.4775	−64.6069	9
26	Belize (2000)	17.0612	−87.8960	17
27	Mexico (2000)	18.4504	−87.4273	3
28	Jamaica (2000)	18.3959	−77.4951	46
29	Cuba (2001)	21.7795	−81.8456	36
30	Cuba (2001)	22.7839	−79.3944	16
31	Cuba (2001)	21.0469	−79.2417	28
32	Panama (2002)	9.4711	−79.5228	28
33	USA (2003)	25.0436	−80.6007	9
34	Puerto Rico (2003)	18.2918	−65.3953	9
35	Nicaragua (2003)	12.1821	−83.0440	5
36	Dominican Republic (2004)	18.7290	−71.0715	8
38	USA (2004)	24.6552	−82.8947	10
39	Dominican Republic (2003)	18.5469	−68.3632	9
			Total	348

Further, given our specific aims, we retained data on four fish groups only. Three of these groups are of high commercial importance and typically used as indicators of fishing pressure, i.e. snappers (8 species), groupers (11 species) and a combination of highly valued commercial species, hereafter commercial spp., classified by AGRRA as “Commercially significant” species in its output fish products, which includes selected species of snappers, groupers, grunts and triggerfishes (21 species, Table S1). The fourth fish group was that of parrotfishes, which here included 12 species recorded (Table S1). Finally, for each fish group, we used data only from reef-surveys in which the specific fish group was present, i.e. reef-surveys in which a particular fish group was not recorded were excluded from the analyses for that fish group. This facilitated interpretation of the comparison among metrics within a fish group, given that estimates of fish average weight would not be available in surveys where fish density and biomass would have been recorded as a zero value. This approach implied that the total number of reef-surveys used in the analyses of each fish group differed somewhat among groups.

#### Indices of fishing pressure

We used two different and independent crude indices of fishing pressure. The first index was based on average human population size within 25 km of a reef-survey site, with this distance representing the likely radius of influence of fishermen [58]. We calculated this index by multiplying the area of land found within a 25 km radius circle centered at the reef site by the average human population density in that land area, yielding an estimate of human population size within 25 km. Human population size has been shown to correlate well with metrics of nominal fishing effort (i.e. boat-meters per km^2^) in the Caribbean and elsewhere [59]. Human population density was obtained from the Gridded Population of the World V.3 as available at the Socioeconomic Data and Applications Center (http://sedac.ciesin.org/gpw/). Human population density estimates for the 1995, 2000 and 2005 periods were strongly correlated (all r_s_>0.99, p<0.001) and therefore averaged to provide a single overall variable for the 1998–2004 period.

The second index was based on information about the effectiveness of protection against fishing at the sites where the fish surveys were carried out, which was derived from independent work by the Reefs at Risk Initiative [7]. For some fish surveys we had more precise information about the management category at the specific time of the survey [60] and so we made use of this information where appropriate. We assigned all 348 fish surveys to either one of 2 management effectiveness categories with respect to fishing: (1) Effective or partially effective management (Full/partial protection), which implies some level of protection against fishing (n = 74 fish surveys), or (2) Unknown or ineffective management or no protection (Unprotected/unknown protection status), which implies lack of protection or lack of knowledge of the protection status (n = 274 fish surveys, Figure 1). The fact that surveys for which we do not know the protection status were pooled with sites with ineffective or no protection will reduce our ability to detect fish metric-fishing pressure relationships if some of these unknown sites do in fact receive some level of protection. In general, the same applies if some surveys are incorrectly allocated to either protection category. However, this should still allow for an informative comparison of the performance of the different fish metrics under the same level of uncertainty. Our two indices of fishing pressure were uncorrelated, i.e. human population size within 25 km did not differ significantly between the two levels of protection effectiveness [two-sample t-test (on rank transformed data): t = 1.3, d.f. = 346, p = 0.169].

### Data Analyses

#### Fish metrics and human population size

We examined potential associations between human population size within 25 km (hereafter human population size) and fish density, average fish weight and fish biomass for each fish group across the region by means of Spearman rank correlation tests. In these analyses, we used only data from the Unprotected/unknown protection category (n = 274 surveys; Figure 1) to minimize the potentially confounding effects of effective protection against fishing. Because we expected decreases in all metrics with increasing human population size, we used one-tailed tests to assess the significance of the correlations at nominal level of 0.05. Further, to account for the spatial autocorrelation present in the data, we ran the tests using a modified correlation test described by Dutilleul et al. [61], which measures the amount of autocorrelation present in the data and adjusts the degrees of freedom of the test accordingly. The spatial layout of the fish surveys was incorportated into the test using their geographic coordinates. Prior to these correlation analyses, all fish metrics and human population size were fourth-root transformed and linearly detrended through multiple regression using each variable as dependent variable and the latitute and longitude coordinates as predictors. These modified correlations were run using the “modttest” package [62] in the R environment [63].

Moreover, for fish density, which is used to derive both fish biomass and average fish weight, we supplemented these correlation analyses by visually examining how the precision of the aforementioned fish density estimates, measured as a coefficient of variation (standard deviation of the ten transects in the fish survey/average of the ten transects in the fish survey), changed with increasing human population size.

#### Fish metrics and effectiveness of protection against fishing

We assessed whether the fish metrics of each fish group differed between protection effectiveness categories across the region using a permutatinal ANOVA (permANOVA). To minimize potentially confounding spatial effects, we carried out all significance tests through permutations restricted within 26 AGRRA sampling batches, i.e. sets of nearby reef-surveys carried out during the same time period, which collectively made up this data-set (Table 2). This implied that the test was effectively testing for differences between protection categories at the location (batch) level. For these analyses, we rank transformed the data in order to reduce the effect of extreme values and minimize heterogeneity of variance, the latter here assessed by a Levene’s test. The permANOVAs were carried out using the “vegan” package [64] in the R environment [63].

#### Parrotfish size and species composition and human population size

We also investigated associations between human population size and average fish weight of individual parrotfish species and parrotfish species composition across the region. We used Spearman rank correlation tests, modified to account for the presence of autocorrelation in the data [61], to assess the significance of associations between human population size and average fish weight of individual parrotfish species. The location of the fish surveys was incorporated into the test using their geographic coordinates. Here, we used data for the eight most frequenly occurring parrotfish species, as the remaning species were too rare to warrant analyses (present in ≤9 fish surveys), and we used only data from fish surveys classifed as Unprotected/unknown protection status (n = 274) to minimize potential confounding effects of fishing protection effectiveness (Figure 1). Prior to these correlation analyses, data from each species were fourth-root transformed and linearly detrended (see above section). To synthesize the results of the correlation analyses across all parrotfish species, we used a fixed-effects meta-analytical approach following Borenstein et al. [65] to produce a single summary correlation value derived from those of the individual species and assess its (one-tailed) significance. We used meta-analytical techniques because this readily allowed us to incorporate the adjusted degrees of freedom from the individual Spearman rank correlations as a weighting factor into the analyses, thus implicitly accounting for the spatial autocorrelation in the data of individual species. The modified correlations were run using the “modttest” package [62] in the R environment [63].

To examine the association between parrotfish species composition and human population size across the region, we performed a Redundancy Analyses (RDA) by constraining the parrotfish species composition matrix (fish density of the individual species as columns and sites as rows) to the human population size vector, while controlling for large scale trends using a matrix with the geographic coordinates of the reef-surveys. The parrotfish matrix was Hellinger-transformed to reduce the influence of extreme values while eliminating the undesirable effect of double zeros on euclidian distances [66]; human population size was fourth-root transformed. Again, we used data from the eight most frequenly occurring parrotfish species and we used only data from reef-surveys classifed as Unprotected/unknown protection status (n = 274; Figure 1). These analyses were carried out using the “vegan” package [64] in the R environment [63].

## Results

### Fish Metrics and Human Population Size

The four fish groups differed in the number of reef-surveys where they were present in both Fully/partially protected sites and sites with Unprotected/unknown protection status (Table 3). Overall, snappers were the least frequently occurring fish group, found in 81% of all the reef-surveys, followed by the groupers (94%), the highly valued commercial spp. (98%) and finally, the parrotfishes, which were the only ubiquitous group (100%; Table 3).

**Table 3:** Summary statistics for fish biomass (grams per 100 m^2^), fish density (fish per 100 m^2^) and average fish weight (grams per fish) for different fish groups across reef-surveys allocated to two categories of protection effectiveness against fishing.

		Unprotected/Unknown protection status (n = 274)				Full/Partial protection (n = 74)			
Metric	Fish group	n	Percent	mean	sd	n	Percent	mean	sd
Fish biomass									
	SNP	226	82	1009.6	1550.2	56	76	824.2	1027.0
	GRP	260	95	280.5	328.8	68	92	373.4	344.7
	COM	269	98	1301.8	1736.4	72	97	1296.3	1272.5
	PAR	274	100	1633.6	1287.3	74	100	1980.0	1365.4
Fish density									
	SNP	226	82	4.5	6.4	56	76	3.8	4.5
	GRP	260	95	1.3	0.9	68	92	1.7	1.2
	COM	269	98	7.2	10.3	72	97	7.2	8.0
	PAR	274	100	15.8	10.4	74	100	12.8	9.4
Average fish weight									
	SNP	226	82	240.9	214.1	56	76	257.7	177.5
	GRP	260	95	247.3	287.0	68	92	295.4	290.5
	COM	269	98	191.2	151.0	72	97	220.4	152.5
	PAR	274	100	116.5	74.7	74	100	215.0	198.4

Snappers (SNP), groupers (GRP), highly valued commercial species (COM) and parrotfishes (PAR). n- number of surveys in which the fish group was present; Percent – percentage of surveys in which the fish group was present; sd-standard deviation. Only data of surveys under the Unprotected/Unknown protection status (left columns) were used in correlation analyses with human population size. See Table S1 for details on species making up these fish groups.

**Figure 2:**
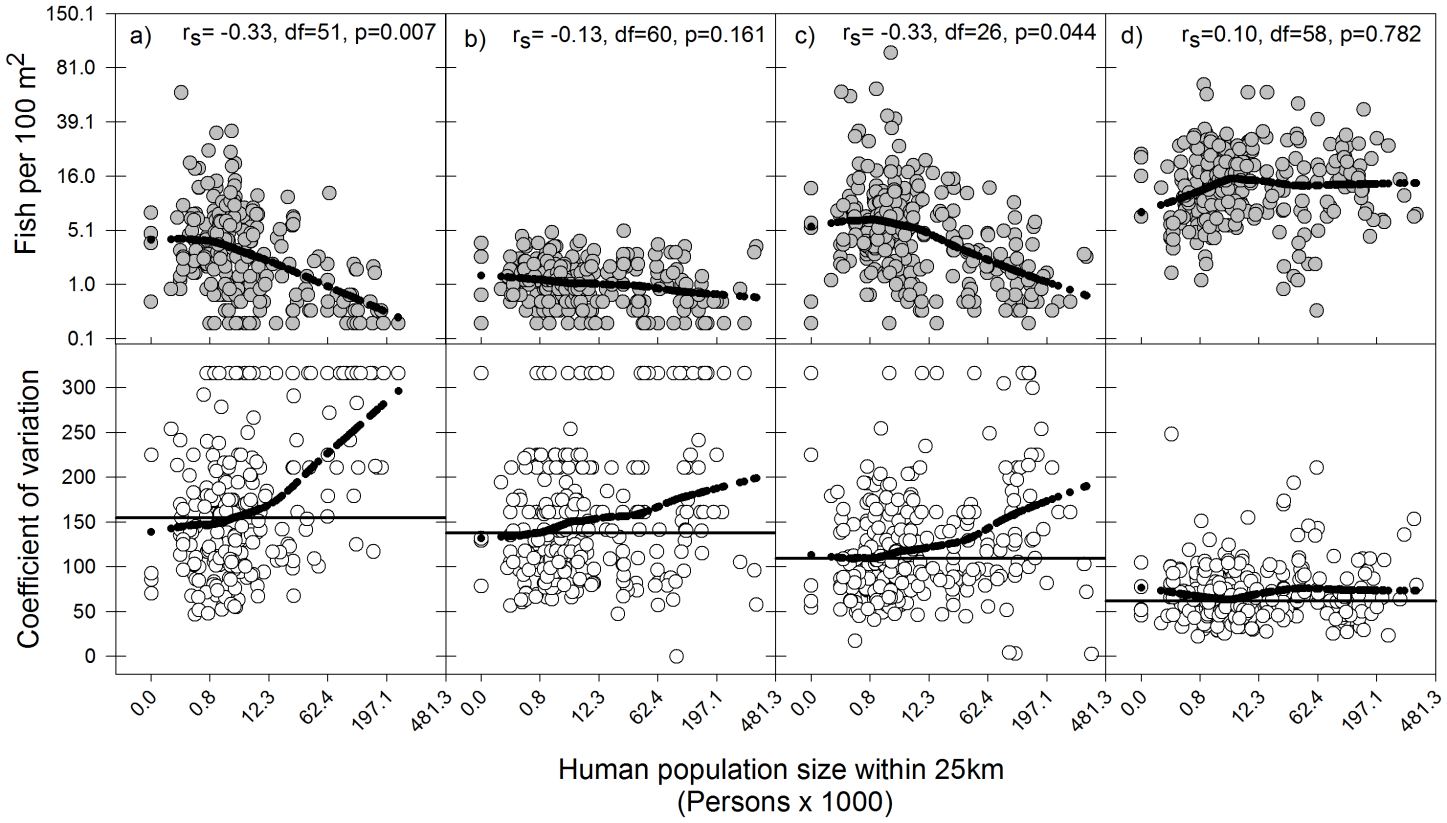
Scatter-plots showing relationships between human population size and fish density for selected fish groups across the Caribbean (top panels) and the coefficient of variation, a measure inversely related to precision, associated with the fish density estimates for each fish group (bottom panels). Selected fish groups are a) snappers (n = 226), b) groupers (n = 260), c) highly valued commercial species (n = 269), and c) parrotfishes (n = 274). Spearman rank correlation coefficients between human population size and the fish metrics are shown, along with the corresponding adjusted degrees of freedom and p-values. Loess smoother black lines were fitted to the data to help visualize trends. Horizontal lines in bottom panels indicate the average coefficient of variation for the fish density estimates of each fish group across all surveys. Fish densities and human population size have been fourth-root transformed before plotting (thus, these axes are plotted on a fourth-root transformed scale), but numbers shown on axes represent back-transformed values. See Table S1 for details on species making up these fish groups.

The relationship between fish density and human population size differed among fish groups. Snappers, groupers and highly valued commercial spp. exhibited negative correlations with human population size and these were significant for both snappers and commercial spp., but not groupers (Figure 2 a, b, c, top panels). However, the latter exhibited a considerably smaller range in fish density values compared to the other two fish groups, which could have contributed to lower the power of the test (Figure 2 a, b, c, top panels). In contrast, parrotfish density exhibited no evidence of a negative association with human population size, as indicated by its small and positive correlation coefficient (Figure 2 d, top panel).

Visual examination of the relationship between human population size and the coefficient of variation (a measure inversely related to precision) of the fish density estimates of the four fish groups also indicated differences between groups. Notably, for snappers, groupers and commercial spp. there was evidence of increases in the coefficient of variation, and hence, decreases in the precision of the fish density estimates with increases in human population size (Figure 2 a, b, c, bottom panels). In contrast to the other fish groups, the coefficient of variation of the estimates of parrotfish density remained relatively uniform across the human population size range (Figure 2 d, bottom panel). Further, parrotfish density exhibited a considerably lower average coefficient of variation than the other three groups, indicating consistent higher precision in the estimates, irrespective of human population size (Figure 2, bottom panels).

**Figure 3:**
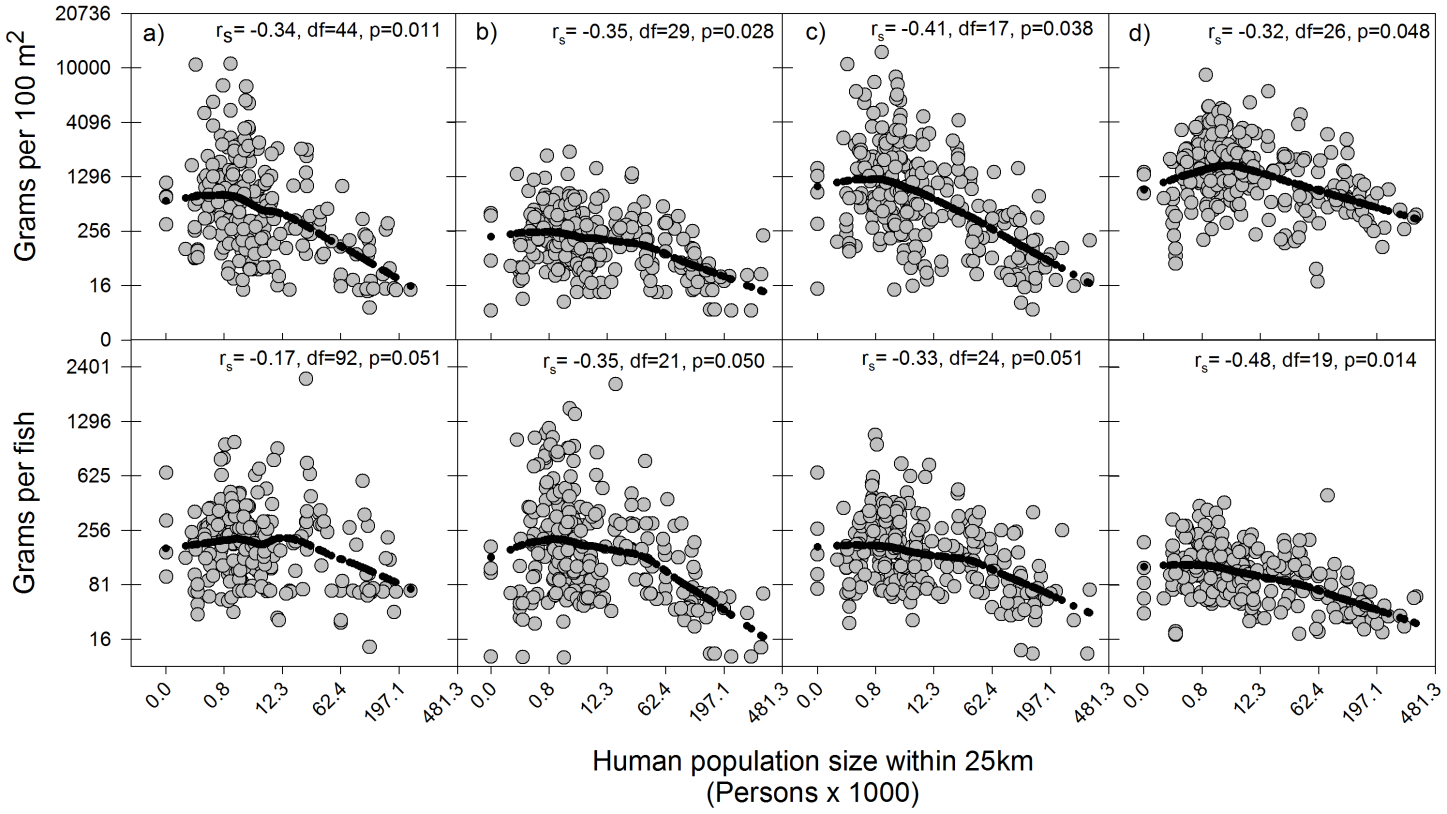
Scatter-plots showing relationships between human population size and fish biomass (top panels) and average fish weight (bottom panels) for selected fish groups across the Caribbean. Selected fish groups are a) snappers (n = 226), b) groupers (n = 260), c) highly valued commercial species (n = 269), and c) parrotfishes (n = 274). Spearman rank correlation coefficients between human population size and the fish metrics are shown, along with the corresponding adjusted degrees of freedom and p-values. Loess smoother black dotted lines were fitted to the data to help visualize trends. All variables have been fourth-root transformed before plotting (thus, all axes are plotted on a fourth-root transformed scale), but numbers shown on axes represent back-transformed values. See Table S1 for details on species making up these fish groups.

In contrast to fish density, all four fish groups were consistent in exhibiting negative correlations between human population size and both fish biomass and average fish weight. These correlations were either significant or marginally significant in all instances (Figure 3). Interestingly, because parrotfish density did not exhibit a negative correlation with human population size (Figure 2 d, top panel), the significant correlation observed for parrotfish biomass must have been driven by average parrotfish weight (Figure 3 d).

Using the magnitude of the correlation coefficient as a crude indicator of the strength of the relationship to compare among all fish metrics, average parrotfish weight outranked all other metrics by exhibiting the strongest relationship with human population size (r_s_ = −0.48, n = 274, p = 0.014). Commercial spp. biomass came second (r_s_ = −0.41, n = 269, p = 0.038), whereas grouper density exhibited the weakest relationship and came last (r_s_ = −0.13, n = 260, p = 0.161), followed by snapper average fish weight (r_s_ = −0.17, n = 226, p = 0.051; Figures 2 and 3). Re-doing the analyses for the biomass and density metrics of the three commercial groups examined, after including the zero values corresponding to sites where no fish were found, also yielded correlation coefficients lower than that of average parrotfish weight (all r≤0.45, n = 274). The latter indicated that the higher correlation coefficient for average parrotfish weight was not an artifact of differences among fish groups in the number of surveys included in the analyses.

### Fish Metrics and Effectiveness of Protection Against Fishing

**Figure 4:**
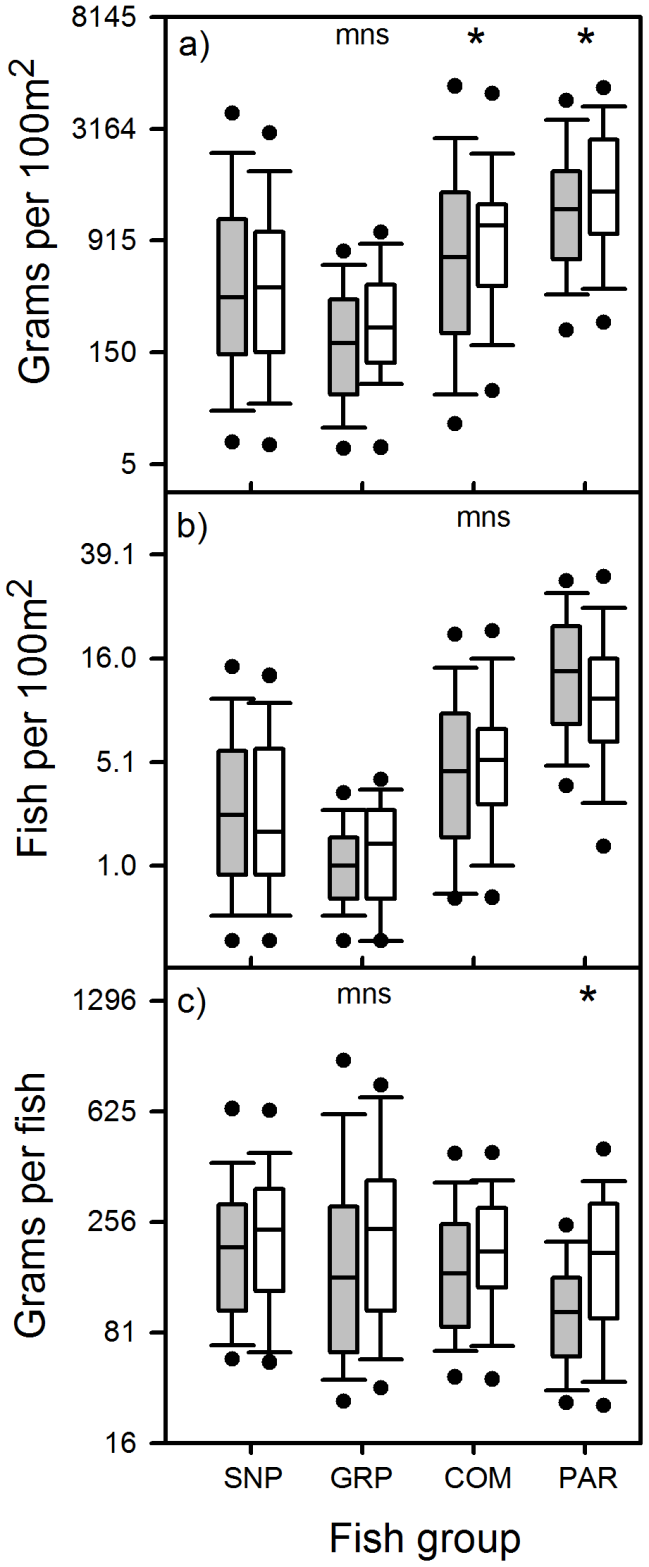
Box-and-whisker plots comparing a) fish biomass, b) fish density and c) average fish weight between reef sites fully/partially protected from fishing (white boxes) and sites unprotected or of unknown protection status (grey boxes) throughout the Caribbean region for snappers (SNP), groupers (GRP), highly valued commercial species (COM) and parrotfishes (PAR). Dots on bottom and top of whiskers represent 5 and 95 percentiles, respectively. All fish metrics have been fourth-root transformed before plotting (thus, vertical axes are plotted on a fourth-root transformed scale), but numbers shown on axes represent back-transformed values. mns- indicates marginally non-significant difference (p<0.1) between protection categories. *-indicates significant difference (p<0.05) between protection categories for a given fish group. See Table S1 for details on species making up these fish groups.

The statistical comparison of protection effectiveness categories across the region [while controlling for the effect of (batch) location] using the different fish metrics for each fish group yielded inconsistent results among metrics and fish groups. We found significant effects (p<0.05) of protection category only for specific fish metrics of certain fish groups. These metrics were commercial spp. biomass, parrotfish biomass and parrotfish average weight, which differed between categories in the expected direction (Table 4, Figure 4). Further, we found marginally significant differences (p<0.1) for commercial spp. fish density and for grouper biomass, which also differed in the expected direction (Table 4, Figure 4).

**Table 4:** Results of PermANOVA comparing fish biomass, fish density and average fish weight for different fish groups between reef-surveys in sites with Full/partial protection against fishing and those in sites with Unprotected/unknown protection status.

		PermANOVA				Levene’s test	
Metric	Fish group	Pseudo-F	df	_adj_R^2^	p-value	F	p-value
Fish biomass							
	SNP	0.0	1, 280	0.000	0.935	0.8	0.358
	GRP	7.6	1, 326	0.020	0.083	1.3	0.260
	**COM**	**2.4**	**1, 339**	**0.004**	**0.045**	**10.6**	**0.001**
	**PAR**	**5.1**	**1, 346**	**0.011**	**0.043**	0.1	0.753
Fish density							
	SNP	0.5	1, 280	0.000	0.837	0.0	0.957
	GRP	3.3	1, 326	0.007	0.160	**11.5**	**0.001**
	COM	0.9	1, 339	0.000	0.064	**6.1**	**0.014**
	PAR	6.6	1, 346	0.016	0.418	0.5	0.460
Average fish weight							
	SNP	1.2	1, 280	0.000	0.163	0.3	0.589
	GRP	6.3	1, 326	0.016	0.079	3.5	0.062
	COM	4.8	1, 339	0.011	0.207	0.7	0.398
	**PAR**	**26.0**	**1, 346**	**0.067**	**0.014**	2.2	0.138

Snappers (SNP), groupers (GRP), highly valued commercial species (COM) and parrotfishes (PAR). To control for spatial effects, permutations were carried out within AGRRA sampling batches (see Table 1). Results of Levene’s test for homogeneity of variance are also shown for each comparison. Bold font indicates significant values at a nominal level of 0.05. See Table S1 for details on species making up these fish groups.

We used the adjusted R^2^ as a crude measure of the strength of the relationship between protection category and metric variability to compare among metrics, as this measure takes into account differences in the number of replicates used in each analysis. Parrotfish average weight considerably outranked all other metrics; it exhibited a six-fold increase in variation explained (adjusted R^2^) relative to the next best metric, i.e. parrotfish biomass (Table 4; Figure 4). However, because parrotfish density overall exhibited an opposite trend to that of average parrotfish weight (Figure 4 b), the significant difference observed for parrotfish biomass is driven by average parrotfish weight (Figure 4). Commercial spp. biomass came in third place, with a negligible amount of explained variation (Table 4). However, this metric exhibited heterogeneity of variance despite rank transformation and so interpretation of this specific result warrants caution. Re-doing the analyses for the biomass and density metrics of the three commercial groups examined, after including the zero values corresponding to sites where no fish were found, did not increase the discriminating power between protection effectiveness categories of these metrics (all adjusted R^2^≤0.010). This confirmed that the better performance of average parrotfish weight was not an artifact of differences among fish groups in the number of surveys included in the analyses.

### Parrotfish Size and Species Composition and Human Population Size

**Figure 5:**
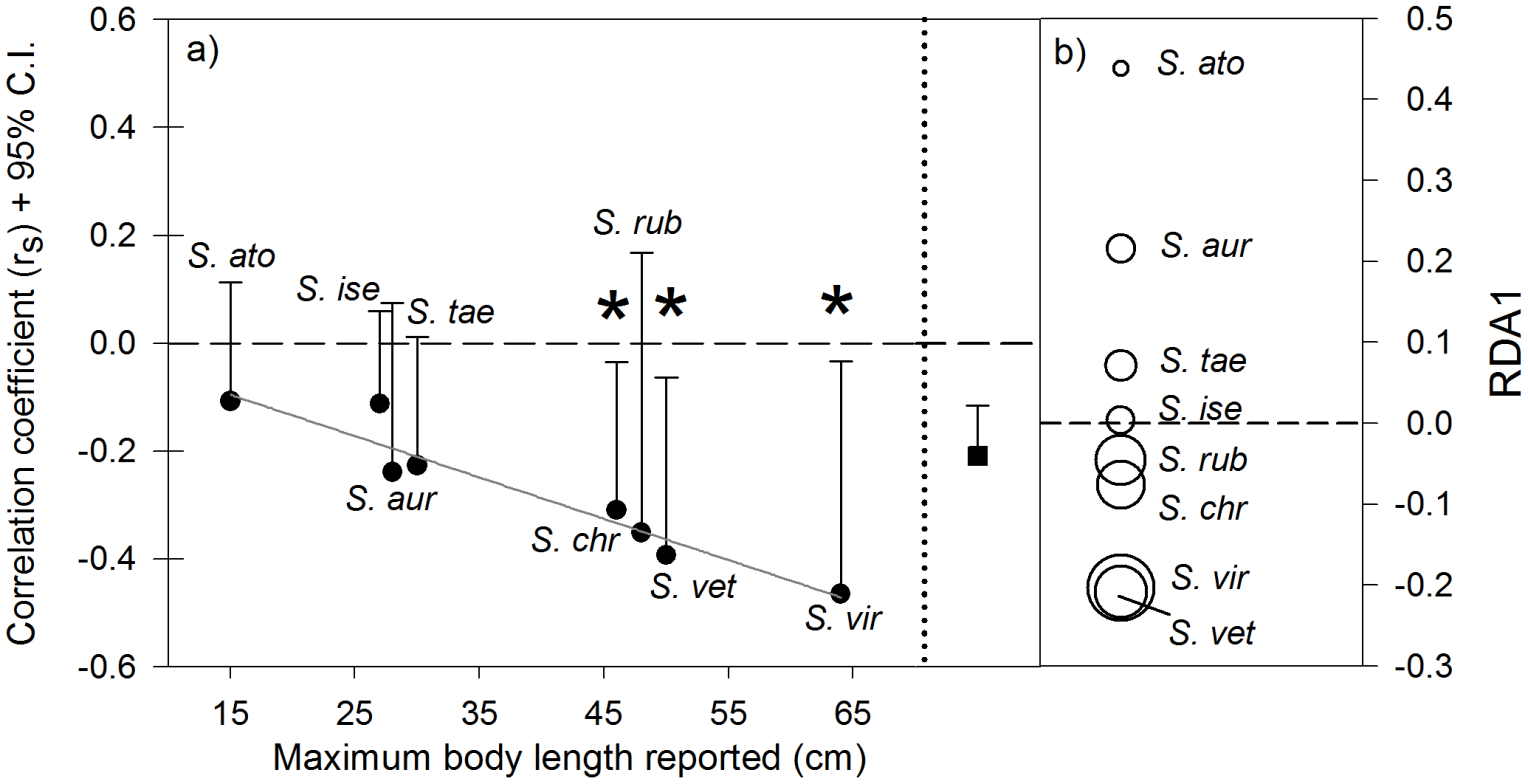
Relationships between human population size and average fish weight and relative fish density of individual parrotfish species across the Caribbean. a) Spearman rank correlation coefficients (+95% one-tailed upper confidence interval; black circles) between human population size and the average fish weight for eight frequently occurring parrotfish species across the Caribbean; the correlation coefficients are ordered as a function maximum body length for each species (as reported by [41]); grey line illustrates the relationship between the magnitude of the correlation coefficients and body size; the eight correlation values were incorporated into one summary correlation coefficient (+95% one-tailed confidence interval; black square); see Table 5 for number of reef-surveys included in the correlation analysis for each species. b) Plot illustrating fish density scores of eight frequently occurring parrotfish species along one redundancy analysis axis representing a gradient of human population size; the species scores were obtained by constraining the species composition of the parrotfish assemblage by human population size across the Caribbean region; the size of the circles representing the species is proportional to the maximum body length of each species (n = 274 reef-surveys).

The eight most frequently occuring parrotfish species in our data-set were the same ones that Hawkins and Roberts [53] found to be most abundant across six Caribbean islands differing markedly in fishing pressure (Table 5). Spearman rank correlations between human population size and the average individual fish weight of each parrotfish species consistently yielded negative coefficients, which were significant (one-tailed p<0.05) for three out of the four largest species examined, i.e. *Sparisoma viride*, *Scarus vetula* and *Sparisoma chrysopterum*, after adjusting for spatial autocorrrelation (Table 5; Figure 5 a). Further, the summary correlation coefficent obtained combining the species-specific correlations through meta-analysis was negative and highly significant (Figure 5 a). Finally, there was a strong negative linear relationship (r = −0.96, n = 8, p<0.001) between the magnitude of such correlation for a species and the maximum size (as body length) attainable by that species (Figure 5 a). In contrast, there was no significant association between the magnitude of the correlation and the number of fish surveys where each species was recorded (r = −0.05, n = 8, p = 0.909), indicating that the strength of the negative association of human population size with fish body size was not an artifact of differences among species in number of fish surveys where they were recorded.

**Table 5:** Summary statistics for average fish weight (grams per fish), fish density (fish per 100 m^2^) and fish biomass (grams per 100 m^2^) of eight frequently occurring parrotfish species across reef-surveys with Unprotected/unknown protection status (n = 274).

	Surveys	Metric					
		Average fish weight		Fish density		Total fish biomass	
Species	n	mean	sd	mean	sd	mean	sd
*Sparisoma aurofrenatum*	270	102.2	62.6	3.5	2.5	340.9	328.0
*Scarus iseri*	266	51.5	34.3	7.7	7.4	356.2	356.2
*Sparisoma viride*	265	250.6	188.7	2.7	2.1	626.4	610.3
*Scarus taeniopterus*	195	103.4	93.3	1.7	2.8	127.3	177.5
*Sparisoma rubripinne*	125	350.0	292.8	0.6	0.7	192.2	271.4
*Scarus vetula*	115	242.3	268.5	1.1	2.1	210.2	396.5
*Sparisoma chrysopterum*	104	332.9	316.2	0.4	0.3	109.7	97.9
*Sparisoma atomarium*	61	14.8	13.7	1.3	1.8	16.4	23.6

n- number of reef-surveys where the species was present; sd-standard deviation. These data were used in species-level correlation analyses with human population size and in the redundancy analysis (RDA) linking parrotfish community composition to human population size. Species are ordered by decreasing order of occurrence across surveys. Note that no species occurred across all the 274 fish surveys.

The RDA examining variability in parrotfish community composition along the gradient in human population size, while controlling for large scale gradients, indicated that 5.8% of the variability in the parrotfish assemblages across the region could be linked to human population size. A plot of the species scores along the single RDA axis (a linear positive function of human population size) revealed a graded arrangement of species based on their body size (Figure 5 b), as indicated by the highly significant negative association between the species scores along the RDA axis and the species maximum attainable body length (r = −0.87, n = 8, p = 0.005). This indicates a negative relationship between human population size and the relative contribution of large parrotfish species to the parrotfish community across the region.

## Discussion

There is great need to improve coral reef fisheries management in the Caribbean in order to ensure the sustainability of the reef fisheries and help reverse current trends of coral reef degradation [2], [12], [13], [20], [67]. The development of simple and intuitive metrics that reliably link the status of the exploited resource to fishing pressure over spatio-temporal scales that are relevant to reef managers represents a fundamental step towards an improved ecosystem-based fisheries management. Given the current depressed state of reef fish populations from overfishing across the Caribbean [7], [8], we hypothesized that metrics derived from fish groups with lower vulnerability to fishing would be more reliable indicators of variability in fishing effects in the region than those from fish groups with higher vulnerability to fishing such as snappers and groupers, which are conventionally and widely used as simple indicators of fishing effects. We argued that the fished, yet ubiquitous, parrotfishes would provide the necessary critical biomass upon which variability in fishing effects could be inferred more consistently and with higher precision throughout the region. Our data support our hypothesis.

This study has demonstrated a consistent and significant relationship between average parrotfish weight and two crude indices of fishing pressure in the Caribbean region. Further, average parrotfish weight consistently outranked any of the metrics derived from conventionally used commercially valuable fish groups with high vulnerability to fishing. Compared to the latter, average parrotfish weight consistently showed a higher sensitivity to indices of fishing pressure across the region as evidenced by (1) a stronger negative correlation with human population size and (2) a stronger (location-specific) positive effect of protection against fishing.

The low sensitivity to fishing pressure of metrics derived from commercial fish groups relative to average parrotfish weight is most likely associated with the consistent lower abundance of these highly vulnerable fish groups across the region (Table 1, Figure 2 a, b, c), due to the pervasive effects of high (historical and current) levels of fishing (e.g. [7]–[9], [38], [68]). It is well known that fishing will have disproportionate effects on the abundance and biomass of fish groups with comparatively larger body-size, slower growth and later maturity [33], [34], [69]. In reefs that are moderately to heavily fished, such as those of the Caribbean, these highly vulnerable fish groups are likely to remain consistently low in abundance even though there might still be considerable variability in fishing pressure. Further, as these highly valued fish groups become rarer due to exploitation, it will become increasingly difficult to obtain sufficient precision in the estimates of fish density and derived aggregate attributes such as average fish size and total fish biomass for any given sampling effort. This effect is clearly demonstrated in our Caribbean-wide analysis. As fish densities of the commercially valuable fish groups decreased with increasing human population size, so did the precision associated with these fish density estimates (Figure 2 a, b c). This supports the idea that the highest uncertainty surrounding the status of these fish groups occurred at the highest levels of exploitation, which is when adopting adequate fisheries management measures and monitoring the expected responses of the fish communities will be most critical [67]. Other fishing-induced factors could have further contributed to lowering the precision of estimates of aggregate attributes for these highly vulnerable fish groups in particular, including active diver avoidance during underwater surveys (e.g. [70]) and enhanced inherent variability in fish abundance (e.g. [71]).

Coincidentally, we suspect that the potential usefulness of some parrotfish metrics as indicators of fishing effects might be partially due to the overall relatively high abundance of parrotfishes across the region (Table 1, Figure 2 d), despite the fact that they are exploited throughout most of their range [42], [43], [48], [53], coupled with the frequent co-existence of multiple parrotfish species with markedly different maximum body sizes at a given location (e.g. [72], [73]). This likely provides sufficient assemblage-level size-structure plasticity and a critical minimum fish biomass upon which the size-dependent effects of fishing can be detected with higher precision. In support of the latter, Hawkins and Roberts [74] compared overall fish biomass of snappers, groupers, surgeonfishes, grunts and parrotfishes across six Caribbean islands with markedly different levels of fishing pressure. Although parrotfish biomass dropped quickly with increasing fishing pressure (albeit not as abruptly as snappers and groupers), it remained consistently higher (up to one order of magnitude) than that of any of the other four fish groups examined across the same fishing pressure gradient [74]. Coincident with this comparatively high overall parrotfish biomass, our own analysis showed that parrotfishes exhibited the highest precision in fish density estimates of all exploited fish groups examined. Importantly, this precision was not associated with variability in human population size, indicating that our ability to make inferences about the status of this fish group holds across the full spectrum of fishing pressure. This consistent higher precision in fish density and derived metrics (i.e. fish biomass and average fish weight) should increase our ability to detect links between parrotfish metrics and fishing pressure in most Caribbean reefs. The limited capacity of fisheries departments across the region to monitor fish community status [31], further highlights the potential value of using parrotfish metrics as simple but cost-effective indicators of anthropogenic drivers on exploited reef fish communities, provided that the appropriate parrotfish metric is selected.

Indeed, our study also showed that the three parrotfish metrics differed considerably in their sensitivity to fishing pressure, with average parrotfish weight outranking biomass and density. This is not surprising given that average parrotfish weight is a size-based metric that seeks to capture the average individual fish size of the parrotfish assemblage in a given location. The potential of size-based metrics applied to fish assemblages as indicators of fishing effects has been highlighted in numerous temperate studies ( [25] and references therein, [56], [57], [75]). The mechanisms underlying the utility of such size-based metrics are well known [25] and include indirect effects of fishing such as the removal of predators and competitors benefiting smaller species [76] as well as direct effects such as the disproportionate removal of the larger individuals of each species [33], [34]. Along a gradient of fishing pressure, such mechanisms would be expected to result in decreases in the relative density of the larger species and/or in a reduction in the average body size of individual species, respectively, as fishing pressure increases. Our study has found evidence for both sets of fishing-induced mechanisms.

Our analysis revealed a change in the relative density of co-occurring parrotfish species, as fishing pressure increased across the Caribbean region, linked to the maximum body size of different parrotfish species. The redundancy analysis (RDA) on the parrotfish assemblage composition showed that the smaller and larger parrotfish species were located at opposite ends of the ordination axis representing the gradient of human population size, with this gradient accounting for approximately 6% of the variability in the relative densities of parrotfish species across the Caribbean region. This amount of explained variance is consistent with that of other studies explaining fish assemblage structure across the Caribbean region (e.g. [77]), considering that we used only a single explanatory variable and that our data spanned multiple locations with markedly different historical and bio-physical factors, which were not directly accounted for in our analysis. Our findings are qualitatively consistent with those of Hawkins and Roberts [53], who compared the densities of seven co-occurring parrotfish species across six Caribbean islands with markedly different historical and current levels of fishing pressure. They found evidence that the absolute density of the larger and smaller species decreased and increased, respectively, along islands constituting an increasing gradient in fishing pressure [53]. Our results are also consistent with those of Clua and Legendre [52] in the South Pacific, who monitored densities of 20 parrotfish species across 5 sites separated by tens to hundreds of km and exhibiting markedly different levels of fishing pressure. They found that the relative density of the larger and smaller parrotfish species decreased and increased, respectively, as fishing pressure increased, with only moderate changes for those species in the intermediate size classes [52]. They concluded that fishing was likely leading to shifts in the dominance of scarid species in their study system [52]. Other studies in the Indo-Pacific [49], [51] and eastern Atlantic [78] have identified declines in the abundance and/or biomass of heavily targeted parrotfish species, which tend to be those with larger maximum body size. Thus, some of the variability in average parrotfish weight across the Caribbean region observed in our study is likely the result of fishing-induced size-dependent shifts in the relative abundance of different parrotfish species, although the correlational nature of this analysis cannot completely exclude the effect of co-varying natural spatial factors (e.g. [79]).

Moreover, our findings support the idea that fishing–induced changes in the average size of individual parrotfish species also play a role in driving variability in average parrotfish weight across the region. There is evidence that fishing reduces the average size of individual co-occurring parrotfish species [50], [51]. In the Caribbean, Hawkins and Roberts [53] compared the average size (as body length) of seven parrotfish species across the six islands with markedly different levels of fishing pressure. They found significant negative relationships between average parrotfish size and fishing pressure for all seven species examined. This is remarkably coherent with our own Caribbean-wide analysis showing consistent negative correlation coefficients between the average fish weight of all individual parrotfish species and human population size; although not all these correlations were significant, their combination unveiled a highly significant overall negative correlation. Furthermore, our analyses indicated that the magnitude of the negative correlation between average fish weight and human population size for a given parrotfish species increased significantly with its maximum body size, as would be expected within any given taxon due to the size-dependent effects of fishing [33], [34].

In contrast to parrotfish average fish weight, parrotfish biomass and density seemed less suitable as potential indicators of fishing effects. In fact, parrotfish density was not linked to any of the indices of fishing pressure in the Caribbean region. This is not necessarily surprising as parrotfish density incorporates the densities of multiple co-occurring parrotfish species that vary in their vulnerability to fishing [48], [49], [51]–[53], with smaller parrotfish species potentially becoming quite abundant in heavily fished areas [53]. This should make this assemblage-level fish metric less sensitive to fishing pressure. Furthermore, overall parrotfish density has been shown to be strongly linked to measures of habitat physical complexity over different spatial extents (e.g. [80]–[82]), suggesting that availability of suitable physical habitat is more important than fishing in explaining overall parrotfish abundance over a range of spatial scales. In support of this, our own preliminary analyses using the same AGRRA data-set (not shown) did reveal a positive and significant correlation between parrotfish density and a measure of habitat physical complexity (i.e. average relief height) across the Caribbean region after accounting for spatial effects (r_s_ = 0.23, n = 274, adjusted d.f. = 90, two-tailed p = 0.024; unpublished data). By extension, because parrotfish biomass is the product of both parrotfish density and average fish weight, it will likely be sensitive to both habitat and fishing effects. This would make parrotfish biomass a less specific, and therefore less suitable, indicator of fishing effects. The latter is supported by our Caribbean-wide analyses, where parrotfish biomass always came second to average parrotfish weight in the associations with indices of fishing pressure, due to the dilution of the fishing signals brought by average parrotfish weight after combining the latter metric with fish density.

The fishing-induced reduction in the average size of individual parrotfish species across the Caribbean region will affect the key and complementary ecological functions that parrotfishes fulfill on the reefs. There is considerable empirical evidence highlighting the importance of algal grazing by large fish herbivores in mediating the outcome of competition for space between algae and corals [17], [19], [54], [83]. This has prompted numerous calls to protect fish herbivores from overfishing, often through the establishment of no-take areas [12], [13], [20]. Parrotfishes play a fundamental role as herbivores due to their different algal feeding modes [13], [84], which complement those of other fish herbivores to effectively control algal growth and enhance coral recruitment and/or survivorship [83]. Parrotfishes also play an important role as reef bio-eroders [55]. Importantly, it is increasingly recognized that the larger parrotfish individuals contribute disproportionately more to algal grazing and bio-erosion than the smaller ones (e.g. [85]–[87]). This underscores the great potential of size-selective fishing to impair such critical ecosystem functions [49], [88] and justifies the need to monitor the status of reef fish herbivores [84]. Our study thus contributes to establishing a critical link between fishing and the integrity of ecological functions through the tangible relationship that both have with average parrotfish size. Further studies could focus on determining the minimum average size of the parrotfish assemblage below which fishing is likely to irreversibly impair the ecological functions performed by parrotfishes, so as to help achieve a balance between harvesting fish and maintaining an ecologically functional fish community on the reef.

Our findings highlight that average parrotfish size deserves further consideration in its ability to infer spatial variability in fishing effects in the Caribbean over a range of spatial scales. This is supported by its significant links with human population size, which varies mainly over broad scales (across states/territories; http://sedac.ciesin.org/gpw/), and with protection effectiveness, which in our analyses was restricted to vary only over local scales (within states/territories). This naturally leads to considering the possibility that average parrotfish size might also be well suited to help infer changes in fishing effects over time (e.g. after enforcement of fishing gear restrictions or no-take marine reserves). There is some evidence that the latter might be the case. Hawkins and Roberts [53] monitored changes in the average size of terminal phase males (as body length) and in total fish biomass for several parrotfish species in St Lucia (West Indies) at yearly intervals, right after the establishment of a network of no-take marine reserves. They noticed a trend of yearly increases in both metrics for most parrotfish species examined, with both metrics also exhibiting consistently greater values inside the marine reserves compared to adjacent fished areas [53]. Similarly, relatively rapid increases in the relative abundance of large parrotfish individuals after cessation of fishing has also been reported in Kenyan reefs, even though such increases might ultimately take decades to level off [89]. This rapid response in key attributes of parrotfish populations after the establishment of fishing protection contrasts with the considerably longer time spans that might be necessary to detect similar changes in other protected target fish groups or species [90], [91]. Thus, average parrotfish size might have a response to fisheries management measures that is detectable over the short periods that are often most relevant for management (e.g. 1–2 years), further highlighting its usefulness as an indicator [21], [92]. This aspect, however, also requires further research.

Some caution is warranted when using size-based metrics as indicators of fishing effects because changes in average size might be responding to environmental factors not directly related to fishing [25]. These potentially confounding factors include density-dependent effects on growth [93] as well as episodes of unusually high recruitment, either of which could contribute to decreasing average fish size in the population of a given species, independently of fishing pressure. We also cannot discard a potentially important role of habitat characteristics, particularly in reef systems where fishing pressure might be low [94]. However, because co-occurring parrotfish species likely differ in key aspects of their population dynamics such as resource use (e.g. [72]) and the timing of recruitment peaks (e.g. [95]), the consistent negative correlations observed between the average fish weight of all parrotfish species and human population size strongly suggests that variability in the assemblage-level average parrotfish size observed here is mainly responding to variability in fishing pressure. This highlights the value of size-based metrics derived from multi-species assemblages as more robust indicators of fishing effects, as they will be less sensitive to species-specific deviations from the fishing-induced trend shared by most species [25]. On the other hand, we cannot discard the possibility that large-scale environmental influences such as climate change might also influence the size-structure of parrotfish communities, although some of the evidence to date in temperate systems suggests that fishing effects on fish community size-structure might still be distinguishable [96]. Finally, where fishing-induced local extinctions of large parrotfish species might have occurred (e.g. *Scarus guacamaia*, [97]), it is possible that effective fisheries management measures might take a very long time to restore parrotfish size-structure to historical levels [98]. Thus, if average parrotfish size, or for that matter, any size-based indicator based on parrotfish assemblages, is used as an indicator of fishing effects, it will be critical to complement and contrast the information it provides with that of a suite of alternative independent indicators of both fishing pressure and resource status [22], [24], [25]. Future research could also focus on exploring the properties of different size-based parrotfish metrics, as they might convey different and complementary information about fishing effects [25].

In summary, our study (1) considerably expands the geographic range in which associations between parrotfish metrics and fishing pressure have been identified in the Caribbean, (2) helps clarify potential mechanisms driving such associations, (3) underscores the potential value of average parrotfish size as a simple alternative indicator of fishing effects for shallow Caribbean reefs, and (4) in doing so, contributes to establish a direct link between fishing and the integrity of key ecological functions that could help set measurable reference values and establish concrete management objectives in the context of ecosystem-based fisheries management in the region.

## Supporting Information

Table S1 **Species list of snappers (SNP), groupers (GRP), commercial spp. (COM) and parrotfishes (PAR) used in the analyses; all species were recorded in at least one of the 348 reef-surveys.** The species included in the commercial spp. group are part of those categorized as “Commercially significant” in the Atlantic Gulf Rapid Reef Assessment (AGGRA) database fish products that were recorded in at least one reef-survey.
(DOCX)
