# Supplementary material for: Parrotfish Size: A Simple yet Useful Alternative Indicator of Fishing Effects on Caribbean Reefs?
Source: PLoS One. 2014 Jan 20;9(1):e86291. doi: 10.1371/journal.pone.0086291 (PMC3896469; doi:10.1371/journal.pone.0086291)
Supplement: Table S1 — Species list of snappers (SNP), groupers (GRP), commercial spp. (COM) and parrotfishes (PAR) used in the analyses; all species were recorded in at least one of the 348 reef-surveys. The species included in the commercial spp. group are part of those categorized as “Commercially significant” in the Atlantic Gulf Rapid Reef Assessment (AGGRA) database fish products that were recorded in at least one reef-survey. (DOCX) [file pone.0086291.s001.docx]

| Common name | Scientific name | Family | SNP | GRP | COM | PAR |
| --- | --- | --- | --- | --- | --- | --- |
| Mutton Snapper | *Lutjanus analis* | Lutjanidae | X |  | X |  |
| Schoolmaster | *Lutjanus apodus* | Lutjanidae | X |  | X |  |
| Cubera Snapper | *Lutjanus cyanopterus* | Lutjanidae | X |  | X |  |
| Gray Snapper | *Lutjanus griseus* | Lutjanidae | X |  | X |  |
| Dog Snapper | *Lutjanus jocu* | Lutjanidae | X |  | X |  |
| Mahogany Snapper | *Lutjanus mahogoni* | Lutjanidae | X |  | X |  |
| Lane Snapper | *Lutjanus synagris* | Lutjanidae | X |  | X |  |
| Yellowtail Snapper | *Ocyurus chrysurus* | Lutjanidae | X |  | X |  |
| Graysby | *Cephalopholis cruentata* | Serranidae |  | X |  |  |
| Coney | *Cephalopholis fulva* | Serranidae |  | X | X |  |
| Rock Hind | *Epinephelus adscensionis* | Serranidae |  | X | X |  |
| Red Hind | *Epinephelus guttatus* | Serranidae |  | X | X |  |
| Jewfish | *Epinephelus itajara* | Serranidae |  | X |  |  |
| Red Grouper | *Epinephelus morio* | Serranidae |  | X | X |  |
| Nassau Grouper | *Epinephelus striatus* | Serranidae |  | X | X |  |
| Black Grouper | *Mycteroperca bonaci* | Serranidae |  | X | X |  |
| Yellowmouth Grouper | *Mycteroperca interstitialis* | Serranidae |  | X | X |  |
| Tiger Grouper | *Mycteroperca tigris* | Serranidae |  | X | X |  |
| Yellowfin Grouper | *Mycteroperca venenosa* | Serranidae |  | X | X |  |
| Black Margate | *Anisotremus surinamensis* | Haemulidae |  |  | X |  |
| French Grunt | *Haemulon flavolineatum* | Haemulidae |  |  | X |  |
| White Margate | *Haemulon album* | Haemulidae |  |  | X |  |
| Ocean Triggerfish | *Canthidermis sufflamen* | Balistidae |  |  | X |  |
| Redband parrotfish | *Sparisoma aurofrenatum* | Scaridae |  |  |  | X |
| Striped parrotfish | *Scarus iseri* | Scaridae |  |  |  | X |
| Stoplight parrotfish | *Sparisoma viride* | Scaridae |  |  |  | X |
| Princess parrotfish | *Scarus taeniopterus* | Scaridae |  |  |  | X |
| Redfin parrtofish | *Sparisoma rubripinne* | Scaridae |  |  |  | X |
| Queen parrotfish | *Scarus vetula* | Scaridae |  |  |  | X |
| Redtail parrotfish | *Sparisoma chrysopterum* | Scaridae |  |  |  | X |
| Greenblotch parrotfish | *Sparisoma atomarium* | Scaridae |  |  |  | X |
| Midnight parrotfish | *Scarus coelestinus* | Scaridae |  |  |  | X |
| Rainbow parrotfish | *Scarus guacamaia* | Scaridae |  |  |  | X |
| Blue parrotfish | *Scarus coeruleus* | Scaridae |  |  |  | X |
| Bucktook parrotfish | *Sparisoma radians* | Scaridae |  |  |  | X |

Note that the ruber jack, *Caranx ruber*, is also considered a “Commercially significant” species by AGGRA, but we did not include this species in our analyses because of its high mobility compared to the reef survey area [Chapman MR, Kramer DL (2000) Movements of fishes within and among fringing coral reefs in Barbados. Environmental Biology of Fishes 57: 11-24]
